# Anaerobic gut fungal community in ostriches (*Struthio camelus*)

**DOI:** 10.1093/ismeco/ycaf144

**Published:** 2025-08-22

**Authors:** Julia Vinzelj, Kathryn Nash, Adrienne L Jones, R Ty Young, Casey H Meili, Carrie J Pratt, Yan Wang, Mostafa S Elshahed, Noha H Youssef

**Affiliations:** Department of Microbiology and Molecular Genetics, Oklahoma State University, Stillwater, OK 74074, United States; Department of Microbiology and Molecular Genetics, Oklahoma State University, Stillwater, OK 74074, United States; Department of Microbiology and Molecular Genetics, Oklahoma State University, Stillwater, OK 74074, United States; Department of Microbiology and Molecular Genetics, Oklahoma State University, Stillwater, OK 74074, United States; Department of Microbiology and Molecular Genetics, Oklahoma State University, Stillwater, OK 74074, United States; Department of Microbiology and Molecular Genetics, Oklahoma State University, Stillwater, OK 74074, United States; Department of Biological Sciences, University of Toronto Scarborough, Toronto, ON M1C 1A4, Canada; Department of Microbiology and Molecular Genetics, Oklahoma State University, Stillwater, OK 74074, United States; Department of Microbiology and Molecular Genetics, Oklahoma State University, Stillwater, OK 74074, United States

**Keywords:** Neocallimastigomycota, transcriptomics, avian gut, ratites, gut mycobiome

## Abstract

Anaerobic gut fungi (AGF; *Neocallimastigomycota*) are crucial for the degradation of plant biomass in herbivores. While extensively studied in mammals, information regarding their occurrence, diversity, and community structure in nonmammalian hosts remains sparse. Here, we report on the AGF community in fecal samples of 13 domesticated ostriches. The ostrich (*Struthio camelus*) is an herbivorous, flightless, hindgut-fermenting member of the class *Aves* (birds). Illumina-based metabarcoding targeting the D2 region of the large ribosomal subunit (28S rRNA) revealed a uniform AGF community with low alpha diversity in the fecal samples. The community was mostly comprised of sequences potentially representing two novel species in the genus *Piromyces,* and a novel genus in the *Neocallimastigomycota*. Sequences affiliated with these novel taxa were absent or extremely rare in datasets derived from mammalian and tortoise samples, indicating a strong pattern of AGF-host association. One *Piromyces* strain (strain Ost1) was successfully isolated. Transcriptomics-enabled molecular dating analysis suggested a divergence time of ≈ 30Mya, a time frame in line with current estimates for ostrich evolution. Comparative gene content analysis between strain Ost1 and other *Piromyces* species from mammalian sources revealed a high degree of similarity. Our findings expand the range of AGF animal hosts to include members of the birds (class *Aves*), highlight a unique AGF community in the ostrich alimentary tract, and document the occurrence of a strong pattern of fungal–host association in ostriches, similar to previously observed patterns in AGF canonical mammalian hosts.

## Introduction

Anaerobic gut fungi (AGF) are a clade of basal, zoospore-producing fungi belonging to the phylum *Neocallimastigomycota* within the subkingdom *Chytridiomyceta* [1]. They inhabit the digestive tracts of herbivores, crucially aiding in the degradation of plant material and its fermentation [2, 3]. To date, 22 genera of AGF have been described [4, 5], though large-scale culture-independent surveys predict at least twice as many still uncultured [6]. AGF were originally isolated from placental mammals [7], and their occurrence and diversity have been extensively studied in domesticated mammalian hosts (e.g. cows, goats, sheep, and horses) owing to their economic importance and ease of sampling [8–14]. Large-scale culture-independent studies, however, have also identified AGF in many additional wild mammalian [6, 15], marsupial [16], and nonmammalian hosts such as green iguanas [17], and tortoises [18]. The recent isolation of novel strains belonging to basal clades of AGF from tortoises highlights the underexplored scope of diversity and host range of AGF [5].

It is currently unclear what exactly defines the AGF ecological niche, though factors such as host phylogeny, herbivory, prolonged feed retention time, and the presence of dedicated fermentation sites within the digestive tract have been proposed as key determinants. Among these, host phylogeny has been shown to have a greater impact on AGF community composition than diet or other environmental factors [6, 17, 19]. AGF are slow-growers and tend to adhere to plant material [20], suggesting that their survival in a competitive environment might, in part, be dependent on longer retention times. This aligns with the mean feed retention times found in ruminants (43–75 h) and mammalian hindgut fermenters (24–47 h), as compared to the retention time found in carnivores [21–24]. Tortoises exhibit the longest retention time (7–14 days) of all AGF hosts identified so far [25–29]. To the best of our knowledge, no comprehensive study has defined the ecological niche of AGF in more detail.

Birds (class *Aves*), a lineage of warm-blooded, nonmammalian vertebrates within the clade *Sauropsida*, exhibit great ecological and physiological diversity. While most extant bird species are omnivorous, an estimated 2% thrive on mostly herbivorous diets [30, 31]. Many of these herbivorous birds compensate for the low digestibility of plant matter by increasing food intake and shortening gastrointestinal retention times [30–32], adaptations that are potentially unfavorable for the establishment of AGF. Ostriches (genus *Struthio*), however, represent an exception within *Aves*. As large, herbivorous, flightless members of the *Palaeognathae* (an infraclass that also includes rheas, cassowaries, emus, and kiwis), they primarily consume grasses, shrubs, and succulents [32, 33]. They are known to be apt lignocellulose degraders, degrading up to 60% of grasses and leaves eaten [34]. They are equipped with highly specialized gastrointestinal adaptations, including the gizzard filled with grit for mechanical disruption and storage of plant biomass, and large, compartmentalized sacculated ceca with an elongated and partly sacculated colon as the main fermentation sites [35]. The retention time in ostriches (30–40 h) resembles that of mammalian hindgut fermenters [36, 37]. Additionally, the ceca and colon are highly efficient in the absorption of water from the digesta, probably an adaptation to the arid environment in which ostriches evolved [36].

We hypothesized that AGF inhabit the alimentary tract of ostriches. To test this hypothesis, we examined the occurrence, diversity, and community structure of AGF communities in ostrich fecal samples using a combination of culture-independent and culture-based surveys. Our results highlight the novelty of AGF taxa encountered in ostriches as well as the differences and similarities compared to their mammalian counterparts. The ecological and evolutionary implications of these findings are discussed.

## Materials and methods

### Samples

Ostrich fecal samples (*n* = 13) from domesticated animals were collected between 2020 and 2022 from the Oklahoma City Zoo, as well as two private ranches in Oklahoma and one in Texas, USA (Table S1). All samples were obtained from adult ostriches, mostly fed a pellet diet composed of corn, soy, and premixed commercially sold ostrich feed. All ostriches included in this study were born in captivity. The samples were obtained shortly after defecation and collected in 15- or 50-ml Falcon tubes that were placed on ice during transfer to the laboratory, where they were stored at −20°C.

Fecal samples were utilized as an approximation of the anaerobic gut fungal community in this study. Fecal collection is noninvasive, painless and risk-free for the animals, and does not require IRB approval. Further, in hindgut fermenters, the colon and caecum chambers are the main sites of fermentation and hence where the majority of AGF biomass is expected to reside. Caeca and colon represent the most distal compartments of the GIT and the last sites before fecal matter is expelled. As such, while we acknowledge that fecal sampling cannot accurately reflect the AGF community in various compartments within an herbivorous alimentary tract; we reason that logistical, ethical, and GIT tract architecture considerations in hindgut fermenters render the utilization of feces as approximation for the AGF community assessment in ostriches appropriate.

### DNA extraction and amplification

DNA extraction was performed using the DNeasy Plant Pro kit (Qiagen®, Germantown, Maryland, USA) according to the manufacturer’s instructions. Plant DNA extraction kits have been adopted by the AGF research community for AGF nucleic acid extraction due to the implementation of harsh treatments targeting plant cell wall. This allows for the breakdown of the highly recalcitrant AGF cell wall [6, 16, 18, 19, 29, 38]. Aliquots from the interior unexposed-to-air portions of fecal samples were taken in an anerobic chamber (Coy Laboratories, Grass Lake, Michigan, US) and used for DNA extraction and isolation (see below). For detection and characterization of the AGF community, the primer pair AGF-LSU-EnvS For and AGF-LSU-EnvS Rev with Illumina overhang adaptors was used [6, 29] to amplify the D2 region of the large ribosomal subunit. DreamTaq 2X master mix (Life Technologies, Carlsbad, California, US) was used for all PCR reactions in this study according to the manufacturer’s instructions. The PCR protocol for all reactions (excluding indexing) consisted of denaturation for 5 min at 95°C followed by 40 cycles of denaturation at 95°C for 1 min, annealing at 55°C for 1 min and elongation at 72°C for 1 min, and a final extension of 72°C for 10 min. Each PCR run also included a nontemplate control to monitor potential contamination. Given that fecal samples are known to produce DNA extracts containing PCR inhibitors [39], additional efforts to obtain amplicons from samples initially showing negative PCR amplification (*n* = 6) included varying the DNA concentrations and DNA to primer ratio.

### Sequencing and sequence processing

PCR cleaning, indexing, and pooling were conducted according to the protocol outlined in [6, 16, 18, 37, 38]. Briefly, PCR products were cleaned using PureLink gel extraction kit (Life Technologies), and indexed using Nextera XT index kit v2 (Illumina Inc., San Diego, CA, USA). Indexed and cleaned products were pooled using the Illumina library pooling calculator (https://support.illumina.com/help/pooling-calculator/pooling-calculator.htm). Pooled libraries were sequenced either at the University of Oklahoma Clinical Genomics Facility (Oklahoma City, Oklahoma, USA) using the MiSeq platform and the 300 bp PE reagent kit in May 2021 (1 sample from the ranches and four samples from OKC Zoo, Table S1). Successful detection of AGF in these original samples promoted the acquisition of additional samples from different locations to expand the data set. This second batch (one sample from OKC Zoo, as well as all other samples from the ranches, Table S1) was sequenced at the Oklahoma State University One Health Innovation Foundation (Stillwater, Oklahoma, USA) using the NextSeq platform and the 300 bp PE reagent kit in 2023. Sequence quality control was conducted as described in [6, 16, 18, 37, 38]. Briefly forward and reverse Illumina reads were assembled using make.contigs command in mothur [40], then screened to remove sequences with ambiguous bases, sequences with homopolymer stretches longer than eight bases, and sequences that were shorter than 200 or longer than 380 bp. Chimeric sequences were detected and removed using chimera.vsearch in mothur.

A two-tier approach [6] was used to assign sequences to previously described genera and candidate genera and to identify novel AGF genera. Genus-level assignments were used to build a shared file (using the mothur commands *phylotype* and make.shared), which was then utilized as an input for downstream analysis.

Confirmatory amplification of the longer D1-D2 LSU fragment (~700 bp) using the primers NL1F (5′-GCATATCAATAAGCGGAGGAAAAG-3′) and GG-NL4 (5′-TCAACATCCTAAGCGTAGGTA-3′) and sequencing using PacBio was conducted on two samples with high proportion of uncultured lineages (shown in boldface in Table S1). These longer reads were not included in the microbial community analysis but were rather utilized to obtain representative sequences of the D1/D2 region of the novel lineages identified but not isolated. Raw reads were processed using PacBio RS_Subreads Protocol and filtered using default settings. Remaining reads were then processed with PacBio RS_ReadsOfInsert Protocol for generating consensus circular sequences (CCs). Mothur was then used to remove any sequence with an average quality score <25, sequences with ambiguous bases, sequences not containing the correct barcode, sequences with >2 bp difference in the primer sequence, and/or sequences with homopolymer stretches longer than 8 bp. We used standalone blastn-short to identify any CCS with the primer sequence in the middle and removed the identified sequences using *remove.seqs*.

Sequences identified as members of the genus *Piromyces* were further binned into species-level operational taxonomic units (OTUs) by assessing percentage divergence patterns to reference cultured *Piromyces* sequences. A cutoff of 3% divergence was used, since it reflects the average divergence between various currently described *Piromyces* species [41, 42]. For ecological distribution analysis, representative sequences of the three most encountered species-level OTUs in ostrich fecal samples were used to query their occurrence in prior broad host diversity surveys [6, 16, 18, 29].

### Alpha diversity

R version 4.4.2 was used for diversity and statistical analyses as explained below. Coverage values were calculated using the command *phyloseq_coverage* in the R package metagMisc [43, 44]. The R package phyloseq (v 1.50.0) [43] was used to calculate alpha diversity estimates (observed, Shannon, Simpson, and inverse Simpson diversity indices) using the command *estimate_richness*. To account for any effect the sample size might have on the results of alpha diversity, we repeated the analysis while subsampling using the size of the smallest sample. Alpha diversity estimates from ostrich datasets were compared to estimates from a subset of mammalian counterparts (25 cattle, 25 goats, 25 sheep, 24 deer, 25 horses) included in a recent study of the mammalian AGF mycobiome [6], as well as to datasets from tortoises obtained in another study (*n* = 11) [18]. The two-sided Wilcoxon signed-rank test for pairwise comparison of means was used to examine the effect of animal species, family, and class on alpha diversity estimates.

### Community structure

The phylogenetic similarity-based weighted UniFrac index (calculated using the *ordinate* command in the phyloseq R package) was used to construct principal coordinate analysis (PCoA) ordination plots using the function *plot_ordination* in the phyloseq R package. The AGF community structure in ostriches was compared to that found in mammalian and reptilian hosts using the same data set used for alpha-diversity comparisons (see above). To partition the dissimilarity among host factors (animal species, family, class, and gut type), we performed PERMDISP tests (using the command *betadisper* in the R package vegan, v2.6–8) [44] followed by running ANOVA tests. Factors that significantly affected the AGF community structure were identified using the ANOVA F-statistics *P*-values, and the percentage variance explained by each factor was calculated as the percentage of the sum of squares of each factor to the total sum of squares.

To identify AGF genera differentially abundant in ostriches, the genus-level shared file created in mothur was used to calculate both linear discriminant analysis (LDA) effect size (LEfSe) and Metastats. Genera with calculated LDA scores and/or significant Metastats *P*-values were considered differentially abundant. Further, to identify AGF community members responsible for the observed community structure in ostriches versus mammalian or reptilian species, Bray-Curtis index values (calculated using the *ordinate* command in the phyloseq R package) were used to construct double principal coordinate analysis (DPCoA) ordination plots using the function *plot_ordination* in the phyloseq R package. To assess ostrich-AGF genera associations, we calculated global phylogenetic signal statistics (Abouheif’s Cmean, Moran’s I, and Pagel’s Lambda) using the *phyloSignal* command in the phylosignal R package (v. 1.3.1) [45], as well as the Local Indicator of Phylogenetic Association (LIPA) using *lipaMoran* command in the phylosignal R package.

### Phylogenetic tree construction

The phylogenetic position of novel AGF genera and species was evaluated by constructing maximum likelihood phylogenetic trees in FastTree [46] based on the MAFFT-generated multiple sequence alignment of the LSU rRNA sequences of the novel taxa to those of all previously reported cultured and uncultured AGF genera (*n* = 67) as references (version 2.0, https://anaerobicfungi.org/databases).

### Enrichment and isolation

Enrichments were set up in an anaerobic chamber (Coy Laboratories, Grass Lake, Michigan, USA) using different substrates (Table 1) in either rumen fluid cellobiose (RFC) [47] or rumen fluid free medium (RFF) [48]. To obtain pure cultures, multiple rounds of subcultivation and roll tubes were conducted. For all enrichments, and subcultures, Balch tubes (18 × 150 mm glass tubes; part number CLS-4209-01; Chemglass Inc., New Jersey, USA) filled with 7 ml broth and sealed with full body butyl rubber stoppers and aluminium crimps were used. For rolltubes, the same tubes were filled with 5 ml of the respective media with the addition of 2% agar (bacteriological grade, Thermo Fisher Scientific, Waltham, Massachusetts, US).

**Table 1:** Enrichments/isolation attempts with ostrich fecal samples targeting *Neocallimastigomycota*.

Species name	Number of enrichments set up	Number of successful enrichments	Media used	Incubation temperatures (°C)	Samples used
*Piromyces* sp. Ost1	7	7	RFC^a^ + SG^c^, RFF^b^ + SG^c^	39, 41, 43	OS_F1, OS_F2
*Piromyces* sp. Ost2	10	0	RFC^a^ + SG^c^, RFF^b^ + SG^c^	39, 41	TY06, TY07, TY08
JV1	6	2	RFC^a^ + C^d^	39, 41	OS_F2

^a^RFC = rumen-fluid cellobiose medium,

^b^RFF = rumen fluid free medium,

^c^+ SG = adding switchgrass as C-source,

^d^+ C = adding cellulose as C-source

Identity of the isolates was determined by amplifying and Sanger-sequencing the D1-D2 region of the LSU rRNA gene using primers NL1F and NL4R (5’-GGTCCGTGTTTCAAGACGG-3′). Sequencing was conducted at the Oklahoma State University Biochemistry and Molecular Biology Core Facility.

### Transcriptomic sequencing and enzymatic potential

An isolate obtained in this study, designated Ost1, was grown in RFC medium to late exponential/early stationary phase (5 days), vacuum-filtered, and total RNA was extracted using the Macherey-Nagel™ NucleoSpin™ RNA mini kit according to the manufacturer’s instructions. RNA-seq was conducted on an Illumina NextSeq 2000 platform using a 2 × 150 bp paired-end library at the One Health Innovation Foundation lab at Oklahoma State University. RNA-Seq reads were quality-trimmed and *de novo* assembled using Trinity (version 2.6.6) [49] with default parameters. Transcripts were clustered with an identity parameter of 95% (−c 0.95) using CD-HIT [50] to remove redundancy. Remaining transcripts were then used for peptide and coding sequence predictions using TransDecoder (version 5.0.2) (Haas, B.J. https://github.com/TransDecoder/TransDecoder) with a minimum peptide length of 100 amino acids. Gene content of *Piromyces* sp. Ost1 transcriptome was compared to nine previously sequenced *Piromyces* transcriptomes [42, 51–53], all isolated from mammals and belonging to six different putative *Piromyces* species. Comparative gene content analysis was carried out via classification of all predicted peptides from all transcriptomes against COG (via BLASTp comparisons against the most updated database at https://ftp.ncbi.nih.gov/pub/COG/COG2020/data/), KOG (via BLASTp comparisons against the most updated database at https://ftp.ncbi.nih.gov/pub/COG/KOG/), and KEGG classification (by running GhostKOALA [54] search on the predicted peptides) schemes.

To examine the CAZymes production potential of *Piromyces* sp. Ost1 compared to other mammalian isolates (*n* = 53) [6, 51–53, 55, 56], as well as tortoise isolates (*n* = 7) [18], we predicted the overall CAZyme content using run_dbcan4 (https://github.com/linnabrown/run_dbcan) to identify glycoside hydrolases (GHs), polysaccharide lyases (PLs), carbohydrate esterases (CEs), alpha amylases (AAs), and carbohydrate-binding motifs (CBMs).

### Phylogenomic analysis and molecular dating

We used the predicted peptides from the *Piromyces* sp. Ost1 transcriptome, as well as the 60 available AGF transcriptomes for phylogenomic analysis and molecular timing of evolutionary divergence [6, 18, 42]. Five *Chytridiomycota* genomes (*Chytriomyces* sp. strain MP 71, *Entophlyctis helioformis* JEL805, *Gaertneriomyces semiglobifer* Barr 43, *Gonapodya prolifera* JEL478, and *Rhizoclosmatium globosum* JEL800) were used as outgroups and to provide calibration points. We used the “fungi_odb10” dataset, including 758 phylogenomic markers for kingdom *Fungi* [57], for our analysis. Profile hidden Markov models (HMMs) of these markers were previously created and used for previous AGF phylogenomic studies [6, 18, 42]. HMMs were used to identify homologues in all AGF transcriptomes, as well as the five *Chytridiomycota* genomes using HMMER3 (http://hmmer.org/). Markers identified with conserved homologs in all datasets were aligned and concatenated for subsequent phylogenomic analyses. IQ-TREE [58] was used to find the best-fit substitution model and to reconstruct the phylogenetic tree with the maximum-likelihood approach. PartitionFinder (v 2.1.1) [59] was used to group the refined alignment and to assign each partition with an independent substitution model. All partition files, along with their corresponding models, were then imported into BEAUti (v 1.10.4) [60] for conducting Bayesian and molecular dating analyses. Two calibration priors were set: a direct fossil record of *Chytridiomycota* from the Rhynie Chert (407Mya) and the emergence time of *Chytridiomycota* (573–770Mya as 95% HPD). We used the Birth–Death incomplete sampling tree model for interspecies relationship analyses. Unlinked strict clock models were used for each partition independently. Three independent runs (30 million generations each) were performed with a default burn-in (10%). Tracer (v1.7.1) [61] was then used to confirm that a sufficiently effective sample size (ESS > 200) was obtained. Finally, TreeAnnotator (v1.10.4) [60] was used to compile the maximum clade credibility (MCC) tree.

### Data availability

Illumina and RNA-seq reads were deposited in NCBI SRA under BioProject accession number PRJNA1231060. Clone sequences of the D1-D2 region of the LSU rRNA from the *Piromyces* sp. Ost1 isolate were deposited in GenBank under accession numbers PV213533-PV213569. PacBio sequence representatives of the *Piromyces* sp. Ost2 and candidate genus JV1 were deposited in GenBank under accession numbers PV226234 and PV226233, respectively.

## Results

### Occurrence and anaerobic gut fungi community composition in ostriches

A total of 342 691 high-quality AGF-affiliated D2-LSU sequences (average per sample 26,361 ± 26 021) were obtained (Table S1). High coverage values, with and without subsampling, indicated that most of the genus-level diversity was captured in all samples (Table S1 and S2).

**Figure 1:**
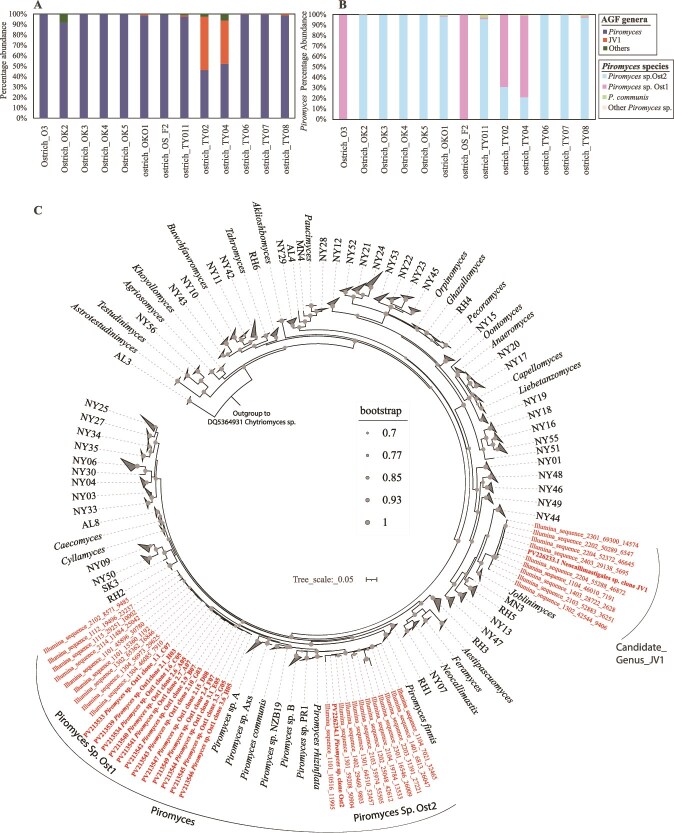
*Neocallimastigomycota* community in ostrich fecal samples. (A) Percentage abundance of AGF genera in ostrich fecal samples. “Others” includes all other genera identified outside of *Piromyces* and the putative novel genus JV1 (detailed in Table S1). (B) Putative species-level affiliation of sequences belonging to genus *Piromyces* in ostrich fecal samples. (C) Phylogenetic tree depicting the position of the two novel *Piromyces* species (*Piromyces* sp. Ost1 and *Piromyces* sp. Ost2) as well as the novel candidate genus JV1 in relation to other cultured and uncultured AGF genera. All reference sequences covered D1/D2 domains of the LSU rRNA gene. The two PacBio-generated sequence representatives of candidate genus JV1 and *Piromyces* sp. Ost2 and all sequences from *Piromyces* sp. Ost1 isolates clones cover D1/D2 domains of the LSU rRNA gene and are shown in bold with GenBank accession numbers. Illumina sequences only cover the D2 region of the LSU rRNA gene.

Phylogenetic analysis indicated that the AGF community in ostriches displayed a high level of similarity and was dominated by sequences affiliated with two genera. Sequences affiliated with the genus *Piromyces* constituted >92% of the community in 11/13 samples and roughly half (44.6 and 51.5%) of the community in the remaining two samples (Fig. 1A, Table S1). The majority of *Piromyces* sequences clustered into two species-level OTUs (Fig. 1B). Both OTUs were phylogenetically distinct from previously named *Piromyces* for which D1-D2 LSU sequence data is currently available (*P. finnis* [62], *P. rhizinflata* [63], and *P. communis* [64]), as well as previously reported and yet to be named isolates *Piromyces* sp. A1 [65], *Piromyces* sp. B4 [65], *Piromyces* sp. NZB19 [66], *Piromyces* sp. PR1 (unpublished, GenBank accession number JN939159), and *Piromyces* sp. Axs (unpublished, GenBank accession number PV351789). As such, the OTUs found in ostriches represent two putative novel *Piromyces* species, for which Ost1 and Ost2 designations are proposed (Fig. 1C). *Piromyces* sp. Ost1 exhibited 96.55% sequence similarity to its closest relative (*Piromyces* sp. A*,* GenBank accession MT085679.1), while *Piromyces* sp. Ost2 exhibited 95.25% sequence similarity to its closest relative (*P. communis* Clone P, GenBank accession ON619893.1) (Table 2). Assessment of the occurrence of these species in prior AGF-focused culture-independent diversity surveys [6, 16, 18, 29] showed that they are either absent or represent only a minor part of the community in the hosts investigated. For example, *Piromyces* sp. Ost1 was completely absent in all mammalian fecal samples examined in Meili *et al.* [6], absent in 12 out of 15 samples examined in Young *et al.* [29], and extremely rare in tortoise (present in one out of 11 samples, constituting 0.02% of total sequences from tortoises) [18] and marsupial (present in two out of 61 samples, constituting 0.001% of total sequences from marsupials) [16] samples. *Piromyces* sp. Ost2 was more frequently encountered in mammalian (404 samples out of 661), marsupial (49 samples out of 61), and tortoise (7 samples out of 11) samples. However, while dominant in ostrich samples, *Piromyces* sp. Ost2 always represented a small fraction of the overall community in other hosts (0.55% of the total community in mammals, 0.24% of the total community in marsupials, and 2.2% of the total community in tortoises) (Table 2).

**Table 2:** Ecological distribution of the ostrich-specific *Piromyces* species and uncultured candidate genus JV1.

Ostrich-specific lineage	Closest cultured representative		Occurrence in previous studies (sequences with% similarity >97%)							
	Genus	% similarity	Mammals [6] (661 samples, 8 772 160 sequences)		Mammals [22] (12 samples, 304 958 sequences)		Marsupials [9] (61 samples, 174 959 sequences)		Tortoises [11] (11 samples, 40 413 sequences)	
			Number	%	Number	%	Number	%	Number	%
*Piromyces* sp. Ost1	*Piromyces*. sp A	96.55	0	0	0	0	2	0.001	8	0.020
*Piromyces* sp. Ost2	*P. communis* clone P	95.25	48 556	0.554	0	0	419	0.239	876	2.168
JV1	*Joblinomyces apicalis*	93.63	762	0.009	0	0	39	0.022	1	0.002

In two out of 13 ostrich samples, roughly half the community encountered was neither affiliated with the genus *Piromyces* nor with any of the currently recognized AGF genera [4, 5] and candidate genera [6, 17, 66]. Rather, it belonged to a monophyletic novel genus-level clade, to which the name JV1 is proposed (Fig. 1A). Sequence divergence within the JV1 clade was low (0.28–1.4%), indicating that all JV1 sequences identified constitute a single species. Candidate genus JV1 is most closely related to the genus *Joblinomyces*, exhibiting 93.63% sequence similarity. Phylogenetic analysis (Fig. 1C) confirmed JV1’s position as member of a clade comprising *Joblinomyces* as well as several yet-uncultured AGF genera (NY44, MN3, RH5, NY13, and NY47) [6, 17, 66]. Assessment of the occurrence of candidate genus JV1 in prior AGF culture-independent diversity surveys with broad host range [6, 16, 18, 29] indicates that JV1 was occasionally encountered (50/661 of mammalian samples, 4/61 of marsupial samples, and 1/11 of tortoise samples). However, like *Piromyces* sp. Ost1 and Ost2, JV1 sequences always represented a very small fraction of the overall community in these hosts (0.009% in mammalian hosts, 0.022% in marsupial hosts, and 0.002% in tortoise hosts) (Table 2).

### Alpha diversity estimates

**Figure 2:**
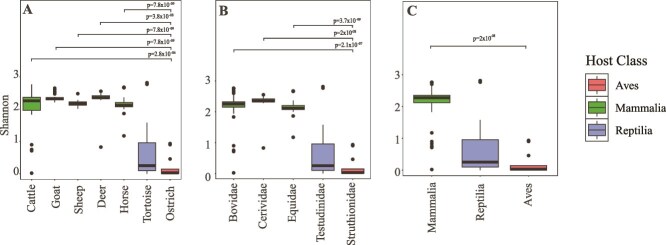
Alpha diversity of *Neocallimastigomycota* in ostriches. Boxplots showing the distribution of Shannon diversity index in ostriches (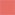) compared to selected mammalian (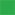) and tortoise (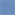) samples. Samples were grouped by animal species (A), animal family (B), and animal class (C). Wilcoxon test *P*-values indicate the significance of differences between ostriches and other mammals. No significant difference (*P* > .05) was identified between ostrich and reptilian samples.

Ostriches harbored an AGF community with low levels of alpha diversity. On average, 10.31 ± 12.53 OTUs were encountered per sample, and values of 0.21 ± 0.34 Shannon index, 0.89 ± 0.2 Simpson, and 1.22 ± 0.46 Inverse Simpson were observed (values are average ± SD from the 13 ostrich samples) (Figs 2 and S2). When accounting for sample size, the number of observed OTUs dropped (3.18 ± 3.16), but alpha diversity estimates did not change (0.20 ± 0.32 Shannon index, 0.89 ± 0.2 Simpson, and 1.22 ± 0.45 inverse Simpson; values are average ± SD from the 13 ostrich samples) (Table S2). These estimates were significantly lower than alpha diversity values in cattle (Wilcoxon *P* < 9.9 × 10^−6^), deer (Wilcoxon *P* < 1.3 × 10^−6^), sheep (Wilcoxon *P* < 1.2 × 10^−5^), goat (Wilcoxon *P* < 9.6 × 10^−7^), and horses (Wilcoxon *P* < 2.8 × 10^−6^), but comparable to values observed in tortoises (Wilcoxon *P* > 0.08), where the AGF community was similarly shown to be dominated by few genera [18] (Fig. 2 and S2).

### Community structure

**Figure 3:**
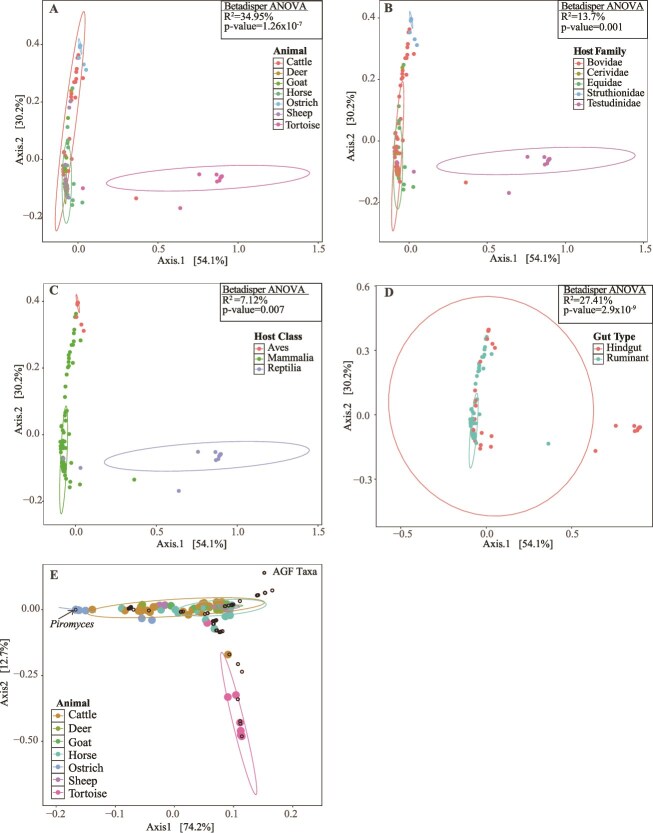
Community structure of *Neocallimastigomycota* in ostriches. (A–D) PCoA plots constructed using weighted UniFrac beta diversity estimates, with a color scheme based on host animal species (A), host family (B), host class (C), and host gut type (D). The % variance explained by the first two axes is displayed on the axes, and results of PERMDISP for the contribution of host factors to the community structure are shown for each plot (R^2^: The % variance explained by each factor, p: F-test *P-*value). (E) Double principal coordinate analysis plot constructed using bray-Curtis beta diversity indices. The AGF taxa are shown as open black circles, and the genus *Piromyces* position is shown with an arrow. Samples are color-coded by the host animal species as in (A).

AGF community structure in domesticated ostriches was compared to that observed in domesticated mammals (cattle, deer, sheep, goat, and horses) as well as tortoises using PCoA. Plots were constructed based on the phylogenetic similarity-based beta diversity index weighted Unifrac (Fig. 3). The first two axes explained 84.3% of the variance. Analysis showed that the animal host species (Fig. 3A), family (Fig. 3B), class (Fig. 3C), and gut type (Fig. 3D) significantly explained 34.95%, 13.7%, 7.16%, and 27.41% of the variance. To identify the specific association between AGF genera and ostriches, double PCoA plots were constructed using Bray-Curtis beta diversity indices (Fig. 3E) and showed the genus *Piromyces* to be associated with ostriches. Similarly, both LEfSe and Metastats analyses showed the genus *Piromyces* to be differentially abundant in ostriches (LEfSe LDA score of 5.6 and *P* = 0, Metastats *P* = .001). In addition, all global phylogenetic signal statistics identified significant correlation between *Piromyces* and ostriches as a host (*P* = .001) (Table S3). Finally, LIPA analysis confirmed the strong significant association between *Piromyces* and ostriches (Table S3) (average LIPA value of 7.82 ± 1.96, *P* = .001). In addition to *Piromyces*, the new uncultured genus JV1 was also differentially abundant in and strongly associated with ostriches (LEfSe LDA score of 4.53 and *P* = 4.6 × 10^−9^, Metastats P = .001), with significant global phylogenetic signal statistics *(P* < .002), and high LIPA values in the two ostrich samples in which it was detected (average LIPA = 5.52 ± 0.055*, P* = .001) (Table S3).

### Enrichments and isolation of anaerobic gut fungi from ostriches

**Figure 4:**
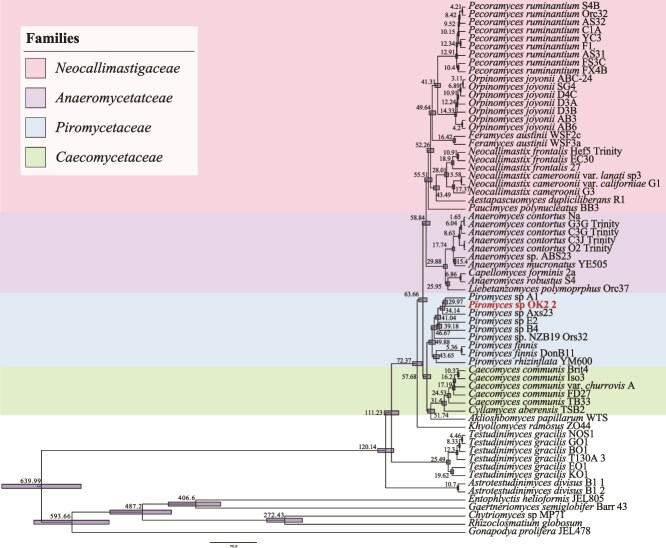
Phylogenomic analysis and molecular timing of strain Ost1. Bayesian phylogenomic maximum clade credibility (MCC) tree of *Neocallimastigomycota* with estimated divergence time for major nodes. Estimate for the divergence time of *Piromyces* sp. Ost1 from its closest mammalian relative (*Piromyces* species A1) is highlighted. The 95% highest probability density (HPD) ranges (horizontal bars) are denoted on the nodes, and the average divergence times are shown.

Multiple enrichments were set up using different media, carbon sources, as well as different incubation temperatures (Table 1). Successful enrichment efforts yielded visible biomass, gas bubbles, and clumping and floating of plant biomass or cellulose, with the identity of AGF determined to be either *Piromyces* sp. Ost1 or candidate genus JV1 by PCR amplification (D1-D2 region of the LSU) and Sanger sequencing. Repeated isolation efforts yielded multiple representatives of *Piromyces* sp. Ost1 (seven strains). Transcriptomic sequencing and subsequent phylogenomic analysis (Fig. 4) confirmed the position of *Piromyces* sp. Ost1 as a novel species within the genus *Piromyces*.

Despite repeated attempts, no pure culture of the candidate genus JV1 from positive enrichments could be obtained. Furthermore, *Piromyces* sp. Ost2 was never enriched (Table 1), despite its predominance in many samples (Fig. 1B).

### Timing the evolution of *Piromyces* sp. Ost1

Transcriptomics-enabled molecular clock timing suggested a divergence time estimate of ≈ 30Mya (95% highest probability density interval of 26.95–32.94Mya) for *Piromyces* sp. Ost1 (Fig. 4). Such time postdates the evolution of the infraclass *Palaeognathae* (~72.8–110Mya) [67, 68], comprising the flightless birds and the volant tinamous, as well as the diversification of *Struthioniformes* and the genus *Struthio* (~69–79.6Mya) [68, 69], but might have coincided with the evolution of flightlessness in these lineages [67].

### Comparative gene content and CAZyome analysis

**Figure 5:**
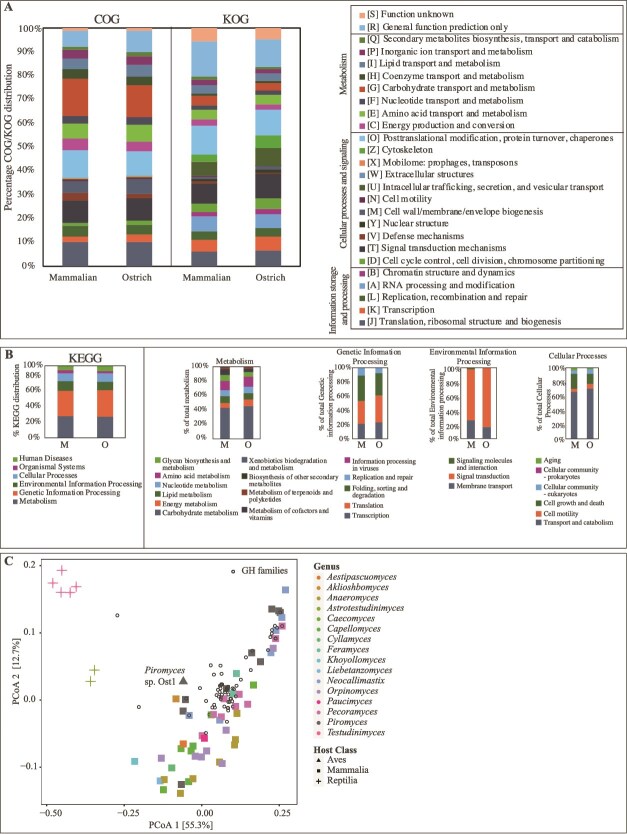
Comparative gene content analysis. (A, B) Gene content comparison between mammalian sourced (M; left stacked columns) and *Piromyces* sp. Ost1 sourced (O; right stacked columns) transcriptomes using COG/KOG (A), and KEGG (B) classification. KEGG classification is further broken down into four main categories: Metabolism, genetic information processing, environmental information processing, and cellular processes. (C) Principal coordinate analysis (PCoA) biplot based on the GH families’ composition in *Piromyces* sp. Ost1 transcriptome (gray triangle) compared to 67 previously obtained AGF transcriptomes belonging to 16 genera (including the genus *Piromyces*). The % variance explained by the first two axes is displayed, and strains are color-coded by AGF genus, as shown in the figure legend. The shapes correspond to the host class, with mammals shown as ■, *Aves* shown as ▲, and reptiles shown as “+.” GH families are shown as empty circles with black borders.

Comparative genomic analysis demonstrated broadly similar COG, KOG, and KEGG profiles between *Piromyces* sp. Ost1 and AGF obtained from mammalian hosts (Fig. 5). In PCoA plots based on GH family composition *Piromyces* sp. Ost1 (Fig. 5C, gray triangle) clustered with the mammalian AGF (Fig. 5C, circles, *n* = 53), and they were both separate from tortoise AGF (*n* = 7), which were previously shown to possess a unique and highly reduced CAZyme repertoire [18]. Overall, comparative gene content and CAZyome analysis suggest functional similarity between ostrich-sourced and mammalian-sourced AGF.

## Discussion

Our investigation of AGF in domesticated ostriches revealed a uniform (Fig. 1A), low diversity (Fig. 2, Table S1, S2) community that was mostly comprised of novel AGF taxa (Fig. 1A and B). The ostrich AGF community was distinct from previously described AGF communities (Fig. 3), with ostrich-associated taxa rarely encountered in mammalian, marsupial, or tortoise datasets (Table 2).

Sequences putatively representing two novel species within the genus *Piromyces* constituted the majority of the AGF community in 11 out of 13 ostrich samples and roughly half the community in the remaining two (Fig. 1A and B). The genus *Piromyces* is ubiquitous, representing an integral member of the AGF diversity in a wide range of mammalian foregut and hindgut fermenters. *Piromyces* was one of the earliest AGF genera to be identified [70], isolated [71], named [64], and characterized [70]. Historically, thallus morphology and flagellation of zoospores were used for taxonomic characterization of AGF isolates, and the clade *Piromyces* comprised any strain with filamentous rhizoids, monocentric thallus development, and monoflagellated zoospores. Currently, the genus *Piromyces* includes all isolates phylogenetically affiliated with the first described monocentric, monoflagellated, and filamentous isolate (*P. communis*) [4, 64, 70–72]. A recent large-scale analysis of available sequencing data for *Neocallimastigomycota* concluded that current members of the genus *Piromyces* display a higher level of within-genus sequence divergence in marker genes (e.g. D1-D2 LSU rRNA ranging from 1.24 to 5.6% with an average of 3.4%), as well as in whole genome metrics (AAI ranging from 72.58 to 99.06% with an average of 79.35%) than that typically encountered within other genera (genus cut-off set at 3% sequence divergence in D1-D2 LSU rRNA and 85% AAI) [4, 42]. While these values support its breakdown into multiple genera, the genus was retained as a single entity [4], partly due to the lack of sequence data from now extinct original type strains for *Piromyces* species (e.g. *P. mae*, *P. dumbonicus*, *P. minutus*, *P. spiralis*, and *P. citronii*) [4, 73, 74].

The two novel, ostrich-associated species encountered in this study exhibited large sequence divergence values from their closest relatives (3.45% and 4.75% D1-D2 LSU sequence divergence for sp. Ost1 and sp. Ost2, respectively). These values would have justified their placement as new genera had they belonged to a different clade/family within the *Neocallimastigomycota*. While it is possible that both novel species identified in this study could belong to previously described *Piromyces* species lacking sequence data, this seems unlikely given that all described species of *Piromyces* have been isolated from mammalian hosts [4], whereas these ostrich-associated species have rarely been identified in mammals (Table 2).

Two out of 13 ostrich samples were dominated by a genus-level cluster (JVI) whose closest relatives (93.63% LSU rRNA sequence similarity) belong to the genus *Joblinomyces* (Table 2, Fig. 1C, Table S1). Both samples came from the same ostrich farm in Texas, USA (Table S1). All samples from this farm contained sequences affiliated with JV1 (0.18–49.65%), while only one sample (out of seven) from other farms/zoos harbored JV1. Given the relatively low number of samples (*n* = 13) and locations (*n* = 4) investigated in this study, an accurate assessment of the general prevalence pattern of candidate genus JV1 in ostriches is not feasible. It is also interesting to note that the two samples with high relative abundance of JV1 had the lowest number of total sequences (*n* = 399 and *n* = 535 for TY02 and TY04, respectively). However, the implication of this observation is currently unclear. Amplicon sequencing provides relative abundance rather than absolute data, and the accurate detection of rare sequences is a central challenge in this approach [75]. Examination of the ecological distribution of candidate genus JV1 in previously published mammalian, marsupial, and reptilian datasets revealed its extremely low abundance (Table 2), hinting that this strain could either be very rare in general or ostrich-specific. A lower absolute AGF sequences load and/or the absence of dominating *Piromyces* sequences could explain the high relative abundance of JV1 in two out of 13 samples. While absolute quantification of AGF sequences in fecal samples using quantitative PCR methods could help elucidate general AGF load in a particular sample, conclusions pertaining to biological implications from such results are inherently limited due to the complex life cycle of AGF, its dependence on the genus and the feeding time of the animals [20], and to variability along the digestive tract [12]. While JV1 could not be isolated in pure culture in this study, it was enriched at two different temperatures (39°C and 41°C) (Table 1), and repeated efforts for its isolation are ongoing.

The AGF community in domesticated ostriches exhibited low levels of alpha diversity, driven by the predominance of one genus (i.e. >50% relative abundance) in most samples. Such a pattern of predominance of one genus has previously been linked to hindgut fermenters in mammals (63% of hindgut fermenters in [6, 16]), and in tortoises (82% of investigated tortoises in [18]), but was less often observed in foregut fermenters (only 25% of foregut fermenters in [6, 16]) (Table S4).

**Figure 6:**
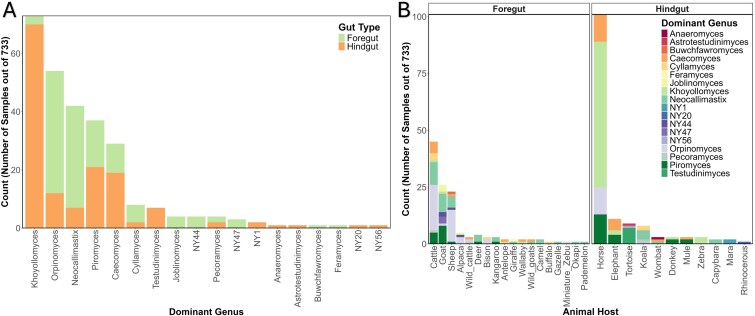
A meta-analysis assessing AGF predominance patterns. Data from three previously published amplicon sequencing studies (targeting the same marker gene region and using the same bioinformatic analysis pipeline) [6, 9, 13] were used to assess patterns of AGF predominance (i.e. a single genus representing >50% of the AGF community) within various hosts. (A) Predominant genera and the gut type they are associated with. (B) the absolute number of samples per animal host that showed a predominance pattern and the AGF genera associated with it. For details on the meta-analysis, refer to Table S3.

While the genus *Piromyces* predominated in ostrich samples in this study, other genera were observed to dominate samples in prior studies. In three studies (a total of 733 samples), the following genera showed relative abundances above 50%: *Khoyollomyces* (*n* = 73), *Orpinomyces* (*n* = 54), *Neocallimastix* (*n* = 42), *Piromyce*s (*n* = 37), and *Caecomyces* (*n* = 29) (Fig. 6A). *Khoyollomyces* and *Caecomyces* primarily dominated mammalian hindgut fermenters (e.g. horses, elephants) (Fig. 6B, Table S4), while *Orpinomyces* and *Neocallimastix* were more common in mammalian foregut fermenters (e.g. cattle, sheep, and goats) (Fig. 6B, Table S4). *Piromyces* showed no clear preference, dominating foregut (*n* = 16) and hindgut (*n* = 21) fermenters (Fig. 6A, Table S4). The reasons underpinning the ecological success of some AGF genera over others are currently unclear. More studies to identify metabolic, physiological, and genomic differences between various AGF taxa, as well as linking such differences to observed host and gut-type preferences of AGF genera are sorely needed to address such issues.

Our results show a clear pattern of host-AGF preference in domesticated ostriches (Fig. 3, Table S3). Our molecular timing analysis estimated an evolutionary time for *Piromyces* sp. Ost1 of ≈ 30Mya. In the context of avian evolution, birds first appeared in the fossil record during the Middle-Late Jurassic (~165–150Mya), diversified by the early Cretaceous, with true modern birds radiating post-Cretaceous and surviving the Cretaceous-Paleogene (K-Pg) extinction event [76]. Within extant birds, the *Palaeognathae* (which includes the flightless ratites and the tinamous) diversified first (72.8–110Mya) [67–69], followed by the diversification of *Struthioniformes* (~69–79.6Mya) [68, 69]. Flightlessness evolved in *Struthioniformes* around 25–30Mya, and the process was tightly associated with the development of herbivory [67]. We therefore propose that the evolutionary timeline of *Piromyces* sp. Ost1 could align with the emergence of flightlessness and herbivory within the ancestors of modern ostriches. This suggests a possible pattern of co-evolution and subsequent retention throughout time up to the evolution of modern ostriches (estimated evolution at 5.3–2.6Mya) [77]. An alternative scenario, where ostrich-specific *Piromyces* sp. Ost1 evolved independently in an unknown host before colonizing modern ostriches after their speciation, cannot be ruled out. However, we deem this scenario less plausible since *Piromyces* sp. Ost1 was rarely identified in animals outside the *Palaeognathae* (mammals or reptiles) and appeared to be specific to the ostrich alimentary tract (Table 2), potential sampling biases notwithstanding.

Finally, to investigate why *Piromyces* sp. Ost1 is highly successful in ostriches but unable to effectively colonize other AGF hosts, we conducted a transcriptomic analysis comparing AGF sourced from different hosts. Comparative gene content analysis showed similar functional profiles (COG, KOG, and KEGG) in *Piromyces* sp. Ost1 compared to mammalian-sourced AGF taxa (Fig. 5A and B). *Piromyces* sp. Ost1 showed a similar CAZyome to mammalian AGF taxa, including mammalian *Piromyces* species (Fig. 5C). While more detailed analysis could clarify finer levels of substrate utilization patterns, the lack of stark differences in broad plant biomass degradation capacities compared to mammalian-sourced isolates is noted. We therefore hypothesize that physiological differences in the ostrich gut (e.g. a slightly higher temperature of 38.1°C–40.5°C) [78, 79] compared to mammalian hindgut fermenters (e.g. 37.5°C–38.5°C in horses) [80] could potentially select for this specific *Piromyces* species. Our preliminary analysis of *Piromyces* sp. Ost1 showed tolerance to higher temperatures and a broader temperature growth range compared to mammalian-sourced *Piromyces* (unpublished data). It is also possible that AGF in the ostrich gut have additional roles beyond plant biomass breakdown, e.g. detoxification, or secondary metabolites secretion, with ostrich AGF specifically adapted to play this role [81, 82]. A more detailed, in-depth experimental and omics-based investigation is needed to address such an interesting question.

In conclusion, this study has expanded the known host range for AGF to include birds (class *Aves*), specifically the common ostrich (*S. camelus*). We demonstrate a strong association between specific AGF taxa and ostriches in the examined samples and indicate a possibility that such pattern could have arisen out of co-evolutionary phylosymbiosis. However, it is important to note that the ostriches investigated in this study were limited to domesticated animals within the south-central part of the USA. Therefore, the role of captivity and pellet-based diet in captivity versus a more varied diet for wild populations remains to be explored. Future studies on wild ostriches, other *Aves* species, as well as other hindgut fermenting animals outside *Mammalia* are needed to confirm the results observed and expand on the global diversity and the evolutionary patterns in *Neocallimastigomycota.*

## Supplementary Material

SupplWithFigure_new_ycaf144

Supplementary_tables_final_new_ycaf144

## Data Availability

The datasets generated and/or analyzed during the study are available through NCBI SRA (BioProject accession number PRJNA1231060) and GenBank (accession numbers PV213533-PV213569, PV226234 and PV226233) as well as in this article and its supplementary information files.
