# Supplementary material for: Anaerobic gut fungal community in ostriches (Struthio camelus)
Source: ISME Commun. 2025 Aug 22;5(1):ycaf144. doi: 10.1093/ismeco/ycaf144 (PMC12423396; doi:10.1093/ismeco/ycaf144)
Supplement: SupplWithFigure_new_ycaf144 [file supplwithfigure_new_ycaf144.pdf]

Supplementary material for:

The anaerobic gut fungal communty in ostriches (*Struthio camelus*)

Julia Vinzelj[1*], Kathryn Nash[1], Adrienne L. Jones[1], R. Ty Young[1], Casey H. Meili[1], Carrie J. Pratt[1], Yan Wang[2], Mostafa S. Elshahed[1], and Noha H. Youssef[1]

[1]Department of Microbiology and Molecular Genetics, Oklahoma State University, Stillwater, OK, USA.

[2] Department of Biological Sciences, University of Toronto Scarborough, Toronto, ON, Canada

Supplementary Tables (provided as an Excel sheet)

Table S1. A list of the 13 samples studied, their sources, the total number of sequences analyzed per sample, sample coverage, and the percentage abundance of AGF genera in each sample.

Table S2. Alpha diversity estimates when subsampling using the sample size of the smallest sample (Ostrich_TY02).

Table S3. Results of Metastats, linear discriminant analysis (LDA) effect size (LEfSe), global, and local phylogenetic signal statistics for the significant association of Piromyces and candidate genus JV1 with ostriches.

Table S4. Comparative analysis of *Neocallimastigomycota* community structure (genus level) in various hosts. The meta analysis is based on the OTU count tables from amplicon sequencing with *Neocallimastigomycota* specific primers targeting the LSU region taken from previously published studies (Meili *et al.* 2023, Jones *et al.* 2024, Pratt *et al.* 2024).

Supplementary figures.

Figure S1. Alpha diversity of *Neocallimastigomycota* in Ostriches. Boxplots showing the distribution of Observed number of genera (A, D, G), Simpson diversity index (B, E, H), and Inverse Simpson (C, F, I) in ostriches (■) compared to selected mammalian (■) and tortoise (■) samples. Samples were grouped by animal species (A-C), animal family (D-F), and animal class (G-I). Wilcoxon test p-values are shown for the significance of difference between ostriches and other mammals. No significant difference ($p > 0.05$) was identified between ostrich and reptilian samples.

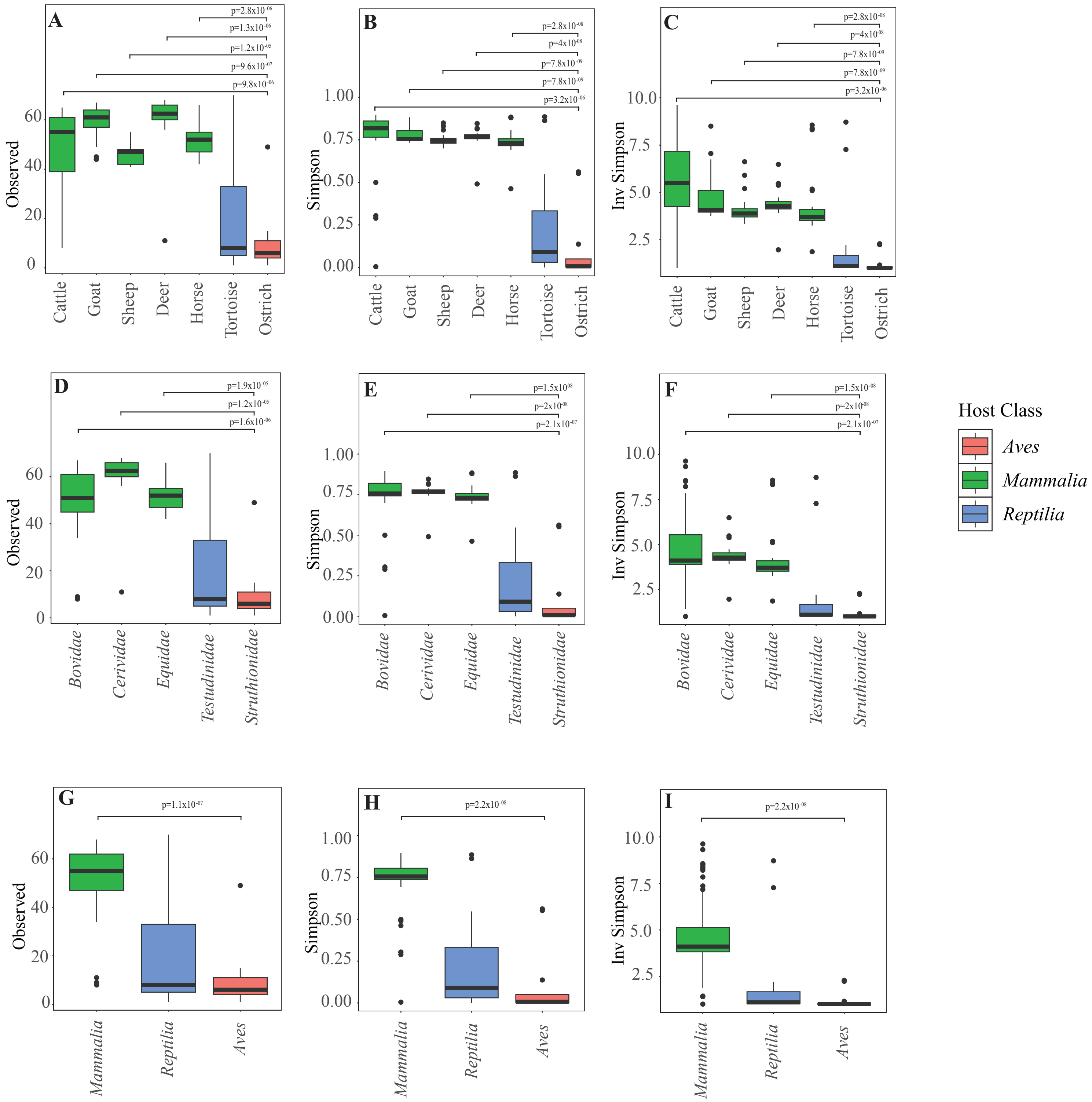
A
Observed
p=2.8x10-06
p=1.3x10-06
p=1.2x10-05
p=9.6x10-07
p=9.8x10-06
0
20
40
60
Cattle
Goat
Sheep
Deer
Horse
Tortoise
Ostrich
B
Simpson
p=2.8x10-08
p=4x1008
p=7.8x10-09
p=7.8x10-09
p=3.2x10-06
0.00
0.25
0.50
0.75
1.00
Cattle
Goat
Sheep
Deer
Horse
Tortoise
Ostrich
C
Inv Simpson
p=2.8x10-08
p=4x1008
p=7.8x10-09
p=7.8x10-09
p=3.2x10-06
2.5
5.0
7.5
10.0
Cattle
Goat
Sheep
Deer
Horse
Tortoise
Ostrich
D
Observed
p=1.9x10-05
p=1.2x10-05
p=1.6x10-06
0
20
40
60
Bovidae
Cerividae
Equidae
Testudinidae
Struthionidae
E
Simpson
p=1.5x1008
p=2x1008
p=2.1x10-07
0.00
0.25
0.50
0.75
1.00
Bovidae
Cerividae
Equidae
Testudinidae
Struthionidae
F
Inv Simpson
p=1.5x10-08
p=2x1008
p=2.1x10-07
2.5
5.0
7.5
10.0
Bovidae
Cerividae
Equidae
Testudinidae
Struthionidae
Host Class
Aves
Mammalia
Reptilia
G
Observed
p=1.1x10-07
0
20
40
60
Mammalia
Reptilia
Aves
H
Simpson
p=2.2x10-08
0.00
0.25
0.50
0.75
1.00
Mammalia
Reptilia
Aves
I
Inv Simpson
p=2.2x10-08
2.5
5.0
7.5
10.0
Mammalia
Reptilia
Aves
